# Impaired plasma clottability induction through fibrinogen degradation by ASP, a serine protease released from *Aeromonas sobria*

**DOI:** 10.1111/j.1574-6968.2008.01184.x

**Published:** 2008-05-06

**Authors:** Takahisa Imamura, Hidetoshi Nitta, Yoshihiro Wada, Hidetomo Kobayashi, Keinosuke Okamoto

**Affiliations:** 1 Department of Molecular Pathology, Faculty of Medical and Pharmaceutical Sciences, Kumamoto University Kumamoto, Japan; 2 Department of Gastroenterological Surgery, Faculty of Medical and Pharmaceutical Sciences, Kumamoto University Kumamoto, Japan; 3 Department of Urology, Faculty of Medical and Pharmaceutical Sciences, Kumamoto University Kumamoto, Japan; 4 Laboratory of Molecular Microbiological Science, Faculty of Pharmaceutical Sciences, Hiroshima International University Hiroshima, Japan; 5 Department of Pharmacogenetics, Faculty of Pharmaceutical Sciences, Okayama University Japan

**Keywords:** *Aeromonas sobria*, sepsis, serine protease, fibrinogen, plasma clotting, bleeding tendency

## Abstract

*Aeromonas sobria* infection often advances to sepsis, in which interaction of bacterial components with plasma proteins possibly causes various disorders. This bacterium releases a serine protease (ASP), a putative virulence factor, and binds to fibrinogen. To study the ASP effect on fibrinogen, we incubated fibrinogen or plasma with ASP and investigated their clotting elicited by thrombin, which converts fibrinogen to a fibrin clot. Enzymatically active ASP retarded plasma clotting in a dose-dependent manner starting at an ASP concentration of 10 nM. ASP also retarded fibrinogen clotting at 3 nM and above, which appeared to correspond to ASP cleavage of fibrinogen at the Aα-chain. Consistent with containing serine protease activity for an ASP-specific substrate, the culture supernatant of an ASP gene-introduced strain retarded plasma and fibrinogen clotting more than that of the wild-type strain. The culture supernatant of an ASP gene-disrupted strain that releases negligible serine protease activity for the ASP-specific substrate did not affect plasma clotting. These results indicate that ASP is the main fibrinogenolytic protease released from *A. sobria*. Impaired plasma clottability induction through fibrinogen degradation is a new virulence activity of ASP and may contribute to hemorrhagic tendencies in sepsis caused by infection with this bacterium.

## Introduction

*Aeromonas* species are facultative anaerobic Gram-negative rods that are widely distributed in aquatic environments (Jones & Wilcox, 1995) and commonly implicated as causative agents of gastroenteritis (Janda & Brenden, 1987; Janda & Abbott, 1998). *Aeromonas* infections, either through wounds or via the digestive tract, often develop into sepsis, particularly in immunocompromised individuals (Janda & Abbott, 1998). *Aeromonas hydrophila*, *Aeromonas sobria*, and *Aeromonas caviae* are three major *Aeromonas* subspecies, and *A. sobria*, frequently isolated from patients' blood (Janda *et al*., 1984), is markedly more virulent than other *Aeromonas* species (Janda & Brenden, 1987).

*Aeromonas* species release several putative virulence factors, including hemolysins, enterotoxins, and proteases (Janda, 1991). As a putative virulence factor, we purified a 65-kDa serine protease, referred to as *Aeromonas* serine protease (ASP), from the culture supernatant of *A. sobria* (Yokoyama *et al*., 2002). ASP is a member of the kexin family of subtilisin-like proteases (Siezen & Leunissen, 1997) and preferentially cleaves peptide bonds at the carboxy-terminal side of paired basic residues (Kobayashi *et al*., 2006). Recently, we found that ASP caused vascular leakage and lowered blood pressure by cleaving components of the plasma kallikrein/kinin system (Imamura *et al*., 2006), suggesting a contribution of ASP to septic shock in *A. sobria* infections. Thus, interaction of ASP with plasma proteins may be associated with the pathophysiology of the infectious disease caused by this pathogen, especially in sepsis.

Fibrinogen, a plasma protein, is converted to a fibrin clot by thrombin, protecting the host from blood loss by bleeding (Mann & Lundblad, 1987). Degradation of fibrinogen and fibrin leads to loss of plasma clottability and rebleeding, respectively, causing hemorrhagic tendencies that are a prominent symptom in disseminated intravascular coagulation (DIC), a common and potentially deadly complication in sepsis patients (Levi, 2001). The ability of *Aeromonas* species to bind fibrinogen (Ascencio *et al*., 1990) suggests that ASP released from *A. sobria* may degrade fibrinogen and abrogate plasma clottability. To examine this virulence activity, we investigated the fibrinogenolytic activity of ASP, and to study the contribution of ASP to fibrinogen degradation by *A. sobria*, we measured the fibrinogenolytic activity of the culture supernatants from an ASP gene-disrupted or -introduced strain.

## Materials and methods

### Materials

Human fibrinogen and bovine thrombin were purchased from Sigma-Aldrich Corp. (St. Louis, MO), and *t*-butyloxycarbonyl–l-glutamyl–l-lysyl–l-lysine–4-methylcoumaryl-7-amide (Boc–Glu–Lys–Lys–MCA) was obtained from the Peptide Institute (Minoh, Japan). Other chemicals were purchased from Wako Pure Chemical Industries (Osaka, Japan). Normal human plasma was prepared by centrifugation of a mixture of 9 vol of freshly drawn blood from healthy volunteers and 1 vol of 3.8% (w/v) sodium citrate. The bacterial strains, plasmids, and their sources used in this study are listed in Table 1. Both *Eschrichia coli* and *A. sobria* were grown at 37 °C in Luria–Bertani (LB) broth and agar plates. A wild-type strain 288 was isolated clinically (Fujii *et al*., 1998).

**Table 1:** Strains and plasmids

	Properties	Sources or references
Strains		
*A. sobria* strains		
288	Wild-type strain producing ASP and metalloprotease	Fujii *et al*. (1998)
288 (Δ*asp*)	288 strain disrupted both *asp* and *orf2*	Khan *et al*. (2007)
T94	Atypical strain not producing ASP and metalloprotease	Khan *et al*. (2007)
*E. coli*		
HB101	Cloning host, *supE44*, Δ(*mcrC-mrr), recA13, ara-14, proA2, lacY1, galK2, rpsL20, xyl-5, mtl-1, leuB6, thi-1*	TaKaRa Co.
SY327λ*pir*	Δ(*lac-pro*) *argE*(Am) *recA*56 *nalA* Rf^r^ (λ*pir*)	Nishibuchi *et al*. (1991)
SM10λ*pir*	*Thi-1 thr leu tonA lacy supE recA*::RP4-2Tc::Mu λ*pir* R6K, Km^r^	Nishibuchi *et al*. (1991)
Plasmids		
Cloning vectors		
pUC119	General cloning vector, Ap^r^	TaKaRa Co.
pSA19CP	Shuttle vector replicable in *E. coli* and *Aeromonas*, Cm^r^	Nomura *et al*. (2002)
pXAC623	Suicide vector, Cm^r^, pEX100T and pKTN701 derivative with R6K *ori* and *mob* RP4	Schweizer & Hoang (1995), Nishibuchi *et al*. (1991)
Constructed plasmids		
pUC119-5528	pUC119 derivative carrying 5528-bp gene fragment containing *asp* and *orf2*	Okamoto *et al*. (2000)
pUC119-5528 (Δ*asp*)	pUC119-5528 defect of the gene fragment from 1776 to 3408 nt	Khan *et al*. (2007)
pXAC-5528 (Δ*asp*)	pXAC623 derivative defect of the gene fragment from 1776 to 3408 nt in 5528-bp gene fragment	Khan *et al*. (2007)
PSA19-5528	pSA19CP derivative carrying 5528-bp gene fragment containing *asp* and *orf2*	Okamoto *et al*. (2000)

### Preparation of *asp*-disrupted or -introduced strain

Fragments of strain 288 chromosomal DNA digested with the EcoRI were inserted into the EcoRI site of pUC119 (Okamoto *et al*., 2000) and introduced into *E. coli* HB101 (TaKaRa Co., Kyoto, Japan). Protease-positive clones were selected using the LB agar medium containing 3% skim milk (Nakarai Co., Kyoto, Japan). Sequence analysis of the cloned insert DNA (5528 bp) in pUC119-5528 (Nomura *et al*., 2002) identified an operon composed of two structural genes, *asp* and *orf2*, which codes for a specific chaperone required for ASP maturation (GenBank accession no. AF253471).

**Fig. 1:**
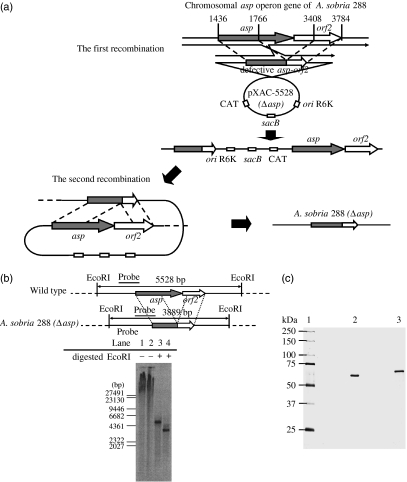
(a) Disruption of *asp*-*orf2* using suicide vector pXAC-5528 (Δ*asp*). The first homologous recombination produced a mutant 288 possessing *asp*-*orf2* and the defective gene on pXAC-5528 (Δ*asp*), and CAT and *sacB* genes. The second homologous recombination occurred between both types of genes located in tandem and produced the *asp*-*orf2*-disrupted strain. (b) Southern-hybridization analysis of *asp*-*orf2*. The *asp*-*orf2* detection DNA probe (b, horizontal bar) that had EcoRI digestion sites at both sides was amplified using two primers AP-20 (5′-CATCGGCGGCAACCGCGGAA-3′) and AP-25 (5′-ATGCCGCTCTCCTTGCCGGT-3′), and labeled digoxigenin DNA Labeling Kit. Total DNAs extracted from both 288 (lanes 1 and 3) and 288 (Δ*asp*) (lanes 2 and 4) using Qiagen Genomic-tips were digested with EcoRI and separated on 0.7% agarose gel. Southern-hybridization reaction with the digoxigenin-labeled probe was performed and hybridized fragments were detected with the digoxigenin luminescent detection kit. Numbers along the left side indicate DNA sizes in base pairs (bp). (C) SDS-PAGE of ASP. ASP (0.6 μg) was analyzed using a SDS-polyacrylamide gel (10%) in the presence (lane 3) or absence of 2-ME (lane 2), and the gel was silver-stained. Lane 1, molecular size markers.

Disruption of *asp* and *orf2* was carried out according to the homologous recombination technique (Kuroda *et al*., 2005) using a suicide vector pXAC623 (Nishibuchi *et al*., 1991; Schweizer & Hoang, 1995). To cleave off the regions from 1766 to 3408, pUC119-5528 was digested with NcoI and BglII and treated with Klenow fragment (TaKaRa Co.). The defective gene fragment was circularized by T4 DNA ligase (TaKaRa Co.) [pUC119-5528 (Δ*asp*)] (Khan *et al*., 2007) and amplified by PCR (2 min at 94 °C, followed by 25 cycles of 10 s at 98 °C, 30 s at 50 °C, and 5 min at 72 °C) using two primers: 5′-TTTCGTTCTAGAGCCGGGCCACGTTCA-3′ and 5′-CGACCCTCTAGAGGGGGCGCCCGGCGG-3′. These primers cover the upstream and downstream regions of the defective gene, respectively, and contain a XbaI site (underlined). The amplified gene fragment was digested using XbaI and inserted into a unique XbaI site of the suicide vector pXAC623. The plasmid, designated as pXAC-5528 (Δ*asp*) (Fig. 1a), was introduced into *E. coli* SM10λ*pir*. The SM10λ*pir* was cultured with strain 288 until the exponential phase and was harvested on a membrane filter (0.2-μm pore size, Advantec Co., Tokyo, Japan) by filtration. This membrane was placed on an LB-broth agar plate and incubated at 37 °C for 3 h to deliver pXAC-5528 (Δ*asp*) from SM10λ*pir* to strain 288 by conjugation. The bacterial cells on the membrane were resuspended in LB broth and cultured at 37 °C for 1 h. The first homologous recombination produced a 288 strain possessing both the wild and defective *asp*-*orf2* genes, and CAT and *sacB* genes from the pXAC623 vector (Fig. 1a). The cell suspension spread onto an LB-broth agar plate containing 5 μg mL^−1^ chloramphenicol and 50 μg mL^−1^ ampicillin was incubated at 30 °C overnight. Only the recombinant strain with the CAT gene grew, and was cultured in LB broth without antibiotics. During this procedure, the second homologous recombination occurred between the full-length and the defective *asp*-*orf2* genes (Fig. 1a). To exclude the strain that did not have the second recombination, therefore retaining the *sacB* gene, the bacterial culture spread onto an LB-broth agar plate containing 10% sucrose was incubated at 25 °C. The *asp*-*orf2*-disrupted 288 strain created, designated as 288 (Δ*asp*), was confirmed by Southern-hybridization analysis (Fig. 1b, lane 4).

To prepare a high ASP secretion strain, strain T94 was introduced with pSA19-5528 containing *asp*-*orf2* and CAT genes (T94/pSA19-5528), and selected using LB-broth agar plates containing 5 μg mL^−1^ chloramphenicol. Strain T94 introduced the vector alone (T94/pSA19CP) was used as a control.

### CFU count

Two hundred microliters of the culture of a strain at the stationary phase was added to 100 mL of LB-broth medium. The medium was incubated at 37 °C with shaking (160 r.p.m.) and 100 μL of the culture sample was taken every 3 h. The sample was serially diluted, plated onto LB-agar plates, and cultured for bacterial colony count.

### Measurement of the protease activity of culture supernatants

The bacterial cell culture supernatant, after a 12-h incubation at 37 °C was centrifuged at 12 000 ***g*** for 15 min. The culture supernatant (35 μL) treated with or without a serine protease inhibitor di-isopropyl fluorophosphate (DFP) (final 1 mM) was added to 65 μL of 1% azocasein in 10 mM Tris-HCl buffer (pH 7.5) and incubated at 37 °C for 1 h, followed by addition of 100 μL of 10% trichloroacetic acid. After centrifugation at 13 000 ***g***, 100 μL of the supernatant was mixed with an equal volume of 1 N NaOH and the A_450 nm_ was measured. The culture supernatant (133 μL) treated with or without DFP (final 1 mM) was added to 12 μL of 1.1 mM Boc–Glu–Lys–Lys–MCA in 11 mM sodium phosphate buffer (pH 7.5) and incubated at 37 °C for 30 min, followed by addition 150 μL of acetic acid (130 mM). The AMC released was measured fluorophotometrically at λ_ex_=380 nm and λ_em_=440 nm using a fluorescence spectrophotometer (Model 650-40, Hitachi Co., Japan).

### Purification of ASP

ASP was purified from the culture supernatant of T94/pSA19-5528 (Okamoto *et al*., 2000) by successive column chromatography. The ASP sample was analyzed by sodium dodecyl sulfate-polyacrylamide gel electrophoresis (SDS-PAGE) (10% polyacrylamide gel) and found to be homogenous, showing a single band under reducing and nonreducing conditions, with a molecular mass of 65 kDa (Fig. 1c). To abolish enzymatic activity, ASP and culture supernatants were treated with 1 mM DFP and dialyzed against 10 mM Tris–HCl (pH 7.3) containing 150 mM NaCl (TBS).

### Thrombin time (TT) assay

Conversion of fibrinogen to fibrin by thrombin leads to clotting; thus, fibrinogen degradation results in impaired clotting. To examine the fibrinogenolytic activity of ASP, the thrombin-induced clotting time prolongation of fibrinogen or plasma incubated with ASP was measured with KC4 (Trinity Biotech, Bray, Ireland). For the TT assay, 67.5 μL of citrated human plasma or fibrinogen (3 mg mL^−1^) was incubated with 7.5 μL of ASP in a plastic cell at 37 °C for 3 min, followed by addition of 75 μL of bovine thrombin (5 U mL^−1^) to initiate clotting. As a control, TBS, used for ASP dilution, was added instead of ASP.

### SDS-PAGE

ASP (15 μL, 300 nM) was incubated with 135 μL of human fibrinogen (3 mg mL^−1^ in 50 mM Tris–HCl, pH 7.4, containing 0.1 M NaCl) at 37 °C. At various time periods, 15 μL of the mixture was withdrawn, followed by the addition of 1 μL of DFP (10 mM) to terminate the reaction. Samples were analyzed by SDS-PAGE under reducing conditions using a 10% polyacrylamide gel. Coomassie Brilliant Blue R-250 was used for protein staining.

## Results

### Reduction of fibrinogen and plasma clottability by ASP

**Fig. 2:**
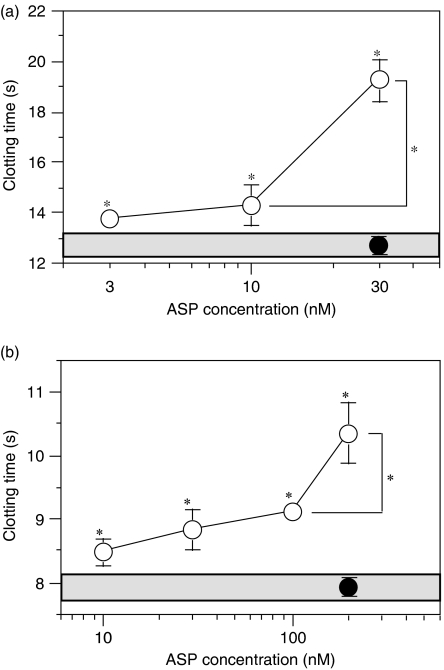
TT prolongation by ASP. For the TT assay, 67.5 μL of fibrinogen (3 mg mL^−1^) (a) or citrated human plasma (b) was incubated with 7.5 μL of ASP in a plastic cell at 37°C for 3 min, followed by addition of 75 μL of bovine thrombin (5 U mL^−1^) to initiate clotting. As a control, TBS was added instead of ASP. ASP concentrations in plasma or fibrinogen solution are shown. Values are expressed as means±SD (*n*=4). ○, nontreated ASP; •, DFP-inactivated ASP. The gray zone indicates the range of TT assayed using TBS instead of ASP. ^*^P<0.01 vs. the control value and between indicated values.

To determine whether ASP could affect fibrinogen clottability, we measured TT after incubating ASP with this clotting factor at the normal plasma concentration (3 mg mL^−1^) (Halkier, 1991). ASP prolonged the fibrinogen TT in a dose-dependent manner, starting at an ASP concentration of 3 nM (Fig. 2a). To examine the ASP effect on fibrinogen under semi-physiological conditions, we measured TT after incubating plasma with ASP. ASP prolonged plasma TT in a dose-dependent manner, starting at an ASP concentration of 10 nM (Fig. 2b), indicating that ASP can reduce the clottability of fibrinogen in plasma. Because DFP-inactivated ASP did not affect TT (Fig. 2a and b), the TT prolongation effect of ASP occurred through its proteolytic cleavage of fibrinogen. Because ASP did not activate plasminogen (data not shown), the involvement of plasmin-mediated fibrinogen degradation in this ASP prolongation effect on thrombin-induced plasma clotting is unlikely. That the clottability reduction of plasma required more ASP than that of fibrinogen (Fig. 2a and b) suggests interference of plasma proteins, including possibly protease inhibitors, in the reaction. Overall, these results indicated that ASP impaired plasma clottability through fibrinogen cleavage.

### Cleavage of human fibrinogen by ASP

**Fig. 3:**
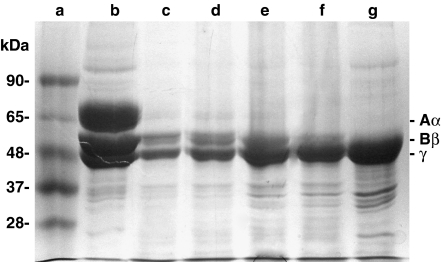
SDS-PAGE analysis of fibrinogen incubated with ASP. Human fibrinogen (3 mg mL^−1^) was incubated with ASP (30 nM) for various time periods and 15 μL of the mixture was withdrawn, followed by addition of 1 μL of DFP (10 mM). Samples were analyzed by SDS-PAGE under reducing conditions using a 10% polyacrylamide gel. Coomassie Brilliant Blue R-250 was used for protein staining. a, markers; b–g, incubated for 0, 2, 4, 8, 16, and 32 min, respectively.

To study the mechanism of the fibrinogen clottability reduction by ASP, fibrinogen was analyzed by SDS-PAGE after incubation with the protease (Fig. 3). ASP degraded fibrinogen in an incubation time-dependent manner and the Aα-chain (66 kDa) disappeared completely at 2 min, followed by Bβ-chain (54 kDa) degradation. Because no clear band, except for the Bβ- and γ-chains, was seen after the Aα-chain disappeared, it seems likely that the Aα-chain was quickly degraded to small peptides. The decrease in the Bβ-chain was coincident with an increase in a band with a molecular weight similar to that of the γ-chain and the appearance of bands smaller than the γ-chain.

### Effect of the culture supernatants of the ASP gene-disrupted or -inserted strain on fibrinogen clottability

**Fig. 4:**
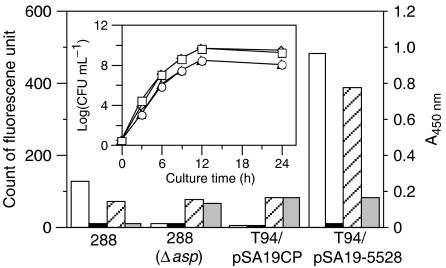
Protease activity of culture supernatants for azocasein (right two bars) or Boc–Glu–Lys–Lys–MCA (10 μL, 10 mM) (left two bars). Open and closed bars indicate the activity of the supernatant treated without or with DFP, respectively. 288, wild strain; 288 (Δ*asp*), *asp*-disrupted strain; T4/pSA19CP, vector-introduced strain; T4/pSA19-5528, *asp* gene-introduced strain. Inset, the time course of growth of strains. The growth of each strain in the culture medium was measured by CFU. □, 288; ⋄, 288 (Δ*asp*); ○, T4/pSA19CP; ▵, T4/pSA19-5528.

To investigate the role of ASP in fibrinogen degradation by *A. sobria*, the culture supernatants of the *asp*-disrupted or the *asp*-introduced strains were examined for their effects on TT. The *asp*-disrupted strain and the wild-type strain grew at almost the same rate, and growth of the T94 strains introduced pSA19-5528 or the vector was slightly lower (Fig. 4, inset). To confirm the effect of *asp* gene modification, we measured the protease activity of culture supernatants using substrates Boc–Glu–Lys–Lys–MCA highly specific for ASP (Kobayashi *et al*., 2006), and azocasein for a broad range of specificities. The supernatant of the *asp*-disrupted strain contained negligible DFP-sensitive protease activity for both substrates, whereas DFP-sensitive protease activity in the supernatant of the *asp*-introduced strain was about fourfold higher than that in the supernatant of the wild strain (Fig. 4). DFP inactivated most of the supernatant protease activity of the wild strain for both substrates and of the *asp*-introduced strain for Boc–Glu–Lys–Lys–MCA, but these supernatants, except for the wild strain supernatant, contained DFP-resistant azocaseinolytic activity at similar levels (Fig. 4). These results indicated that ASP accounted for almost all of the serine protease activity released from the wild strain and was absent in the culture supernatant of the *asp*-disrupted strain.

**Fig. 5:**
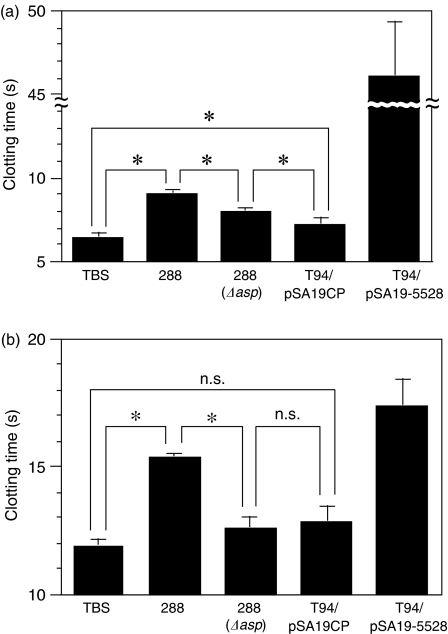
TT prolongation by culture supernatants. For the TT assay, 67.5 μL of human fibrinogen (4 mg mL^−1^) (a) or 52.5 μL of citrated plasma (b) was incubated with 7.5 or 22.5 μL, respectively, of the supernatants in a plastic cell at 37°C for 2 min, followed by addition of 75 μL of bovine thrombin (5 U mL^−1^) to initiate coagulation. Values are expressed as means±SD (*n*=4). ^*^P<0.01; NS, not significant.

The culture supernatants prolonged fibrinogen TT; in terms of this effect, the wild-type strain was much lower than the *asp*-introduced strain, but higher than the *asp*-disrupted strain (Fig. 5a). Although the *asp*-disrupted strain did not secrete ASP (Fig. 4), its culture supernatant prolonged fibrinogen TT significantly (Fig. 5a), apparently indicating the effect of non-serine-type fibrinogenolytic protease(s) in the supernatant (Fig. 4). The supernatant of the vector-introduced T94 strain, secreting neither ASP nor metalloprotease (Table 1), also had a significant fibrinogenolytic activity lower than that of the wild strain (Fig. 5a). The culture supernatant of the *asp*-introduced strain prolonged human plasma TT the most, followed by that of the wild-type strain; culture supernatants of the *asp*-disrupted and the vector-introduced strains exerted no significant effect on plasma TT (Fig. 5b), consistent with the order of the serine protease activity for the ASP-specific substrate in the supernatants (Fig. 4). Based on the DFP-sensitive serine protease activity for the ASP-specific substrate, the wild-strain culture supernatant was estimated to contain about 170 nM ASP, corresponding to 17 nM in the TT assay. The TT-prolongation effect of the wild-type strain supernatant (Fig. 5a and b) appeared to be close to that of 17 nM ASP (Fig. 2a and b), and slightly higher, probably because of the effect of other proteases in the supernatant. Taken together, ASP likely accounted for most of the fibrinogen clottability-abrogating activity secreted from the wild *A. sobria* and caused impaired plasma clotting.

## Discussion

Invading bacteria interact with host components, causing infectious diseases. Plasma proteins leak from the blood stream into infected sites through vascular permeability, enhanced by inflammatory responses against infections, and in sepsis, bacteria flow in the plasma. It is relevant that the interaction of plasma proteins with bacterial components is involved in the pathophysiology of infectious disease. In fact, the interaction of ASP with human fibrinogen resulted in rapid degradation (Fig. 3) and clottability reduction (Fig. 2a and b) of this clotting factor. The *asp*-disrupted strain culture supernatant, deficient in ASP (Fig. 4), lacked most of the TT prolongation activity, whereas that of the wild-type or the ASP gene-introduced strain exerted this activity in a serine protease-dependent manner (Fig. 5a and b), indicating a major contribution of ASP to the fibrinogenolytic activity released from *A. sobria*. Furthermore, the finding that the culture supernatant of the *A. sobria* wild-type strain impaired plasma clottability (Fig. 5b) suggests the induction of hemorrhagic tendencies due to fibrinogen degradation by ASP secreted from this pathogen in sepsis. A subtilisin-like serine protease from group B *Streptococcus* has been reported to cleave human fibrinogen and the fact that the protease-gene-null mutant was 10 times less virulent in a neonatal rat sepsis model of group B *Streptococcus* infection indicates that this fibrinogen-cleaving protease is an important virulence factor of this pathogen (Harris *et al*., 2003). Fibrinogenolysis by ASP is thought to be a new virulence activity of *A. sobria*, in addition to the kinin-releasing activity associated with shock induction (Imamura *et al*., 2006).

Estimating from the previous results (Imamura *et al*., 1995), the result that the fibrinogen TT was prolonged by ASP at 30 nM (Fig. 2b) indicated that more than 80% of the fibrinogen lost clottability after a 3-min incubation with ASP, corresponding to the disappearance of the Aα-chain by 2 min (Fig. 3). The rapid cleavage of the fibrinogen Aα-chain by ASP (Fig. 3), like the physiological fibrinogenolytic protease plasmin (Pizzo *et al*., 1972), indicates ASP's preference for this chain as a substrate. ASP is a subtilisin-like protease that prefers paired Lys and/or Arg residues and cleaves the peptide bonds at the carboxy-terminal side of the paired basic amino acid residues (Kobayashi *et al*., 2006). That such paired residues are fewer in the γ-chain (two pairs) than in the Aα- and the Bβ-chains (seven pairs) (Halkier, 1991) may be associated with the γ-chain being relatively resistant to ASP, but is not consistent with ASP degrading the Aα-chain faster than the Bβ-chain (Fig. 3). The ability of ASP to cleave the peptide bonds at the carboxy-terminal side of Arg residues (Imamura *et al*., 2006; Kobayashi *et al*., 2006) and differences in the three-dimensional structure between the Aα- and Bβ-chains may explain the ASP cleavage preference for the Aα-chain (Fig. 3). Although ASP is the major fibrinogenolytic protease in the culture supernatant of the wild-type *A. sobria* strain, significant fibrinogenolytic activity remains in the *asp*-disrupted strain culture supernatant (Fig. 5a), even though it contains negligible serine protease activity (Fig. 4). *Aeromonas* species produce metalloproteases (Toma *et al*., 1999), and the culture supernatant of the vector-introduced T94 strain, which does not produce ASP and metalloprotease (Khan *et al*., 2007), also contained DFP-resistant caseinolytic activity comparable to that of the *asp*-disrupted strain (Fig. 4). Nonserine proteases are likely involved in the residual fibrinogenolytic activity in the culture supernatant of the *asp*-disrupted strain. However, the result that culture supernatants of the *asp*-disrupted or the vector-introduced T94 strains exerted no significant effect on plasma TT (Fig. 5b) suggested a marginal effect of other proteases on the plasma clottability. Taken together, fibrinogen cleavage at the Aα-chain by ASP appears to be primarily responsible for plasma clottability abrogation by *A. sobria*.

This study demonstrates that ASP, secreted by *A. sobria* at the infection sites or in the circulation, can cause fibrinogen degradation as a major executioner, possibly contributing to induction of hemorrhagic tendencies in sepsis. Accordingly, ASP is considered to be an important virulence factor; thus, it may be a therapeutic target for *A. sobria* infection diseases and ASP-specific inhibitors may be useful as drugs. *Aeromonas* species other than *A. sobria* also secrete subtilisin-type serine proteases (64 kDa) (Coleman & Whitby, 1993), and their infections also cause sepsis (Janda & Brenden, 1987; Janda *et al*., 1994). Thus, such inhibitors may be widely beneficial in the treatment of diseases caused by *Aeromonas* infections.

## Statement

Reuse of this article is permitted in accordance with the Creative Commons Deed, Attribution 2.5, which does not permit commercial exploitation.
